# *Mycobacterium tuberculosis* reactivates latent HIV-1 in T cells *in vitro*

**DOI:** 10.1371/journal.pone.0185162

**Published:** 2017-09-26

**Authors:** Erica C. Larson, Camille L. Novis, Laura J. Martins, Amanda B. Macedo, Kadyn E. Kimball, Alberto Bosque, Vicente Planelles, Louis R. Barrows

**Affiliations:** 1 Department of Pharmacology and Toxicology, University of Utah, Salt Lake City, Utah, United States of America; 2 Department of Pathology, University of Utah, Salt Lake City, Utah, United States of America; Fundació Institut d’Investigació en Ciències de la Salut Germans Trias i Pujol, Universitat Autònoma de Barcelona, SPAIN

## Abstract

Following proviral integration into the host cell genome and establishment of a latent state, the human immunodeficiency virus type 1 (HIV-1) can reenter a productive life cycle in response to various stimuli. HIV-1 reactivation occurs when transcription factors, such as nuclear factor-κB (NF-κB), nuclear factor of activated T cells (NFAT), and activator protein -1 (AP-1), bind cognate sites within the long terminal repeat (LTR) region of the HIV-1 provirus to promote transcription. Interestingly, pattern recognition receptors (PRRs) that recognize pathogen-associated molecular patterns (PAMPs) can reactivate latent HIV-1 through activation of the transcription factor NF-κB. Some PRRs are expressed on central memory CD4^+^ T cells (T_CM_), which in HIV-1 patients constitute the main reservoir of latent HIV-1. *Mycobacterium tuberculosis* (Mtb), the causative agent of tuberculosis (TB), interacts with PRRs through membrane components. However, the ability of Mtb to reactivate latent HIV-1 has not been extensively studied. Here we show that phosphatidylinositol mannoside 6 (PIM6), a component of the Mtb membrane, in addition to whole bacteria in co-culture, can reactivate HIV-1 in a primary T_CM_ cell model of latency. Using a JLAT model of HIV-1 latency, we found this interaction to be mediated through Toll-like receptor-2 (TLR-2). Thus, we describe a mechanism by which Mtb can exacerbate HIV-1 infection. We hypothesize that chronic Mtb infection can drive HIV-1 reactivation. The phenomenon described here could explain, in part, the poor prognosis that characterizes HIV-1/Mtb co-infection.

## Introduction

Tuberculosis is the leading cause of death for individuals living with human immunodeficiency virus type-1 (HIV-1) [1–4]. In 2015 alone, it was estimated that one in every three deaths among HIV-1-infected individuals was due to TB [2]. HIV-1-infected individuals are ~20–30 times more likely to contract TB compared to uninfected individuals [5]. HIV-1/Mtb co-infected patients exhibit accelerated HIV-1 disease and shorter overall survival [6]. In addition, the risk for active TB infection increases from around 10% in a lifetime to 10% per year for patients that are co-infected with HIV-1 [3]. This accelerated disease progression indicates an interaction between these two pathogens.

*Mycobacterium tuberculosis* (Mtb) is the causative agent of TB. HIV-1 has been proposed to exacerbate Mtb pathogenesis via multiple mechanisms. Evidence of this interaction includes: granuloma disorganization and reduced bacterial containment due to HIV-1 replication at sites of Mtb infection [7]; impaired phagosomal killing of Mtb in alveolar macrophages of HIV-1/Mtb co-infected patients [8]; HIV-1 infection decreases CD4^+^ and CD8^+^ T cell counts within granulomas [9]; and HIV-1 alters the function and phenotype of Mtb-specific T cells [10, 11].

Mechanisms by which Mtb interacts with HIV-1 have also been investigated. It is well established that Mtb enhances HIV-1 production and infectivity [12–16]. Toossi and colleagues showed that regions of the lung involved in Mtb infection (i.e. granulomas) contain higher levels of HIV-1 Gag p24 and increased reverse transcriptase activity when compared to uninvolved regions [17]. Mtb-triggered inflammation causes localization of HIV-1-infected cells to these sites of inflammation [7]. Mtb promotes HIV-1 trans-infection while suppressing major Histocompatibility Complex Class II antigen processing by dendritic cells [18]. Different clinical strains of Mtb differentially upregulate HIV-1 production in *ex vivo* peripheral blood mononuclear cells (PBMCs) [19]. Mtb co-infection induces HIV-1 expression in a transgenic mouse model [15]. Furthermore, mycobacterial components signaling via TLR-2 accelerate viral production in co-stimulated T cells [20]. In this work, we describe a new mechanism by which these two pathogens might synergize: Mtb-induced latency reversal in HIV-1 infected central memory T cells (T_CM_).

Microbial infections are often sensed by the innate immune system via host-expressed pattern recognition receptors (PRRs) [21, 22]. PRRs recognize many classes of molecules characteristic of infectious agents including nucleic acids, proteins, lipids, and carbohydrates [23]. Among PRRs, Toll-like receptor (TLRs) are present on the cell surface (TLR-1,2,4,5,6,10) or within endosomes (TLR-3,7,8,9) that recognize such pathogen-associated molecular patterns (PAMP) [24]. TLRs are common on immune system cells including: dendritic cells, macrophages, granulocytes, T cells, B cells, NK cells and mast cells [23].

Previously, Novis et al. demonstrated the ability of TLR-1/2 agonists to reactivate latent HIV-1 *in vitro* [25]. The synthetic lipopeptide TLR1/2 agonist, Pam3CSK4, lead to reactivation of latent HIV-1 in cultured T_CM_ cells from healthy donors, and also in CD4^+^ T cells from aviremic HIV-1 patients. It was shown that the transcription factors NF-κB, NFAT and AP-1 cooperated to induce viral reactivation downstream of TLR-1/2 stimulation. Recently, the mycobacterial membrane component, phosphatidylinositol mannoside 6 (PIM6), was shown to accelerate HIV-1 viral production in co-stimulated CD3^+^ T cells through TLR-2 activation [20]. In the present study, we describe the ability of whole Mtb (H37Ra) and *Mycobacterium smegmatis* in co-culture, H37Rv lysate and PIM6, but not lipoarabinomannan (LAM; a component of the bacterial cell wall), to reactivate latent HIV-1 in JLAT 10.6 (JLAT) cells and in a cultured human T_CM_ model of HIV-1 latency [25–29]. These data support the hypothesis that Mtb, in part through TLR-2, activates pro-inflammatory pathways to enhance transcription of latent HIV-1 during co-infection.

## Results

### PIM6 and H37Rv lysate activate HIV-1 reporter GFP in TLR-2-overexpressing JLAT 10.6 cell

**Fig 1:**
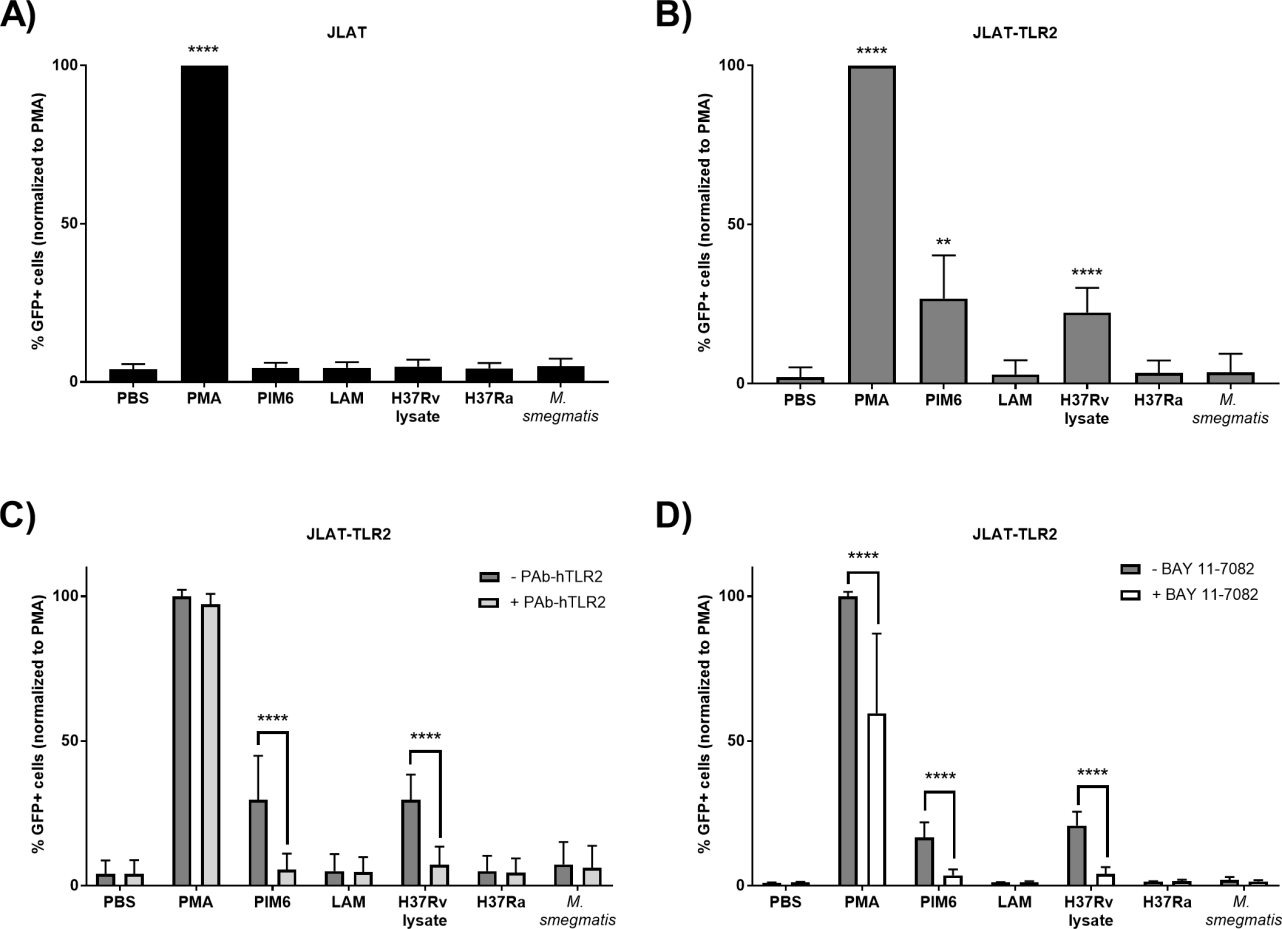
PIM6 and H37Rv lysate induce GFP expression through TLR-2. A) JLAT cells were incubated for 16 hours and GFP expression was measured by flow cytometry. Data represent mean ± SD of four independent experiments run in triplicate. *p<0.05 compared to PBS control. B) JLAT-TLR2 cells were incubated for 16 hours and GFP expression was measured by flow cytometry. Data represent mean ± SD of ten independent experiments run in triplicate. *p<0.05 compared to PBS control. **C**) JLAT-TLR2 cells were pre-incubated with the TLR-2 neutralizing antibody, PAb-hTLR2, for 30 minutes prior to addition of test conditions. Cells were subsequently incubated for 16 hours and GFP expression was measured by flow cytometry. Data represent mean ± SD. *p<0.05 test condition in the absence of TLR-2 neutralizing antibody compared to test condition in the presence of TLR-2 neutralizing antibody. D) JLAT-TLR2 cells pre-incubated with BAY 11–7082 for 30 minutes prior to addition of conditions. Cells were subsequently incubated for 16 hours and GFP expression was measured by flow cytometry. Data represent mean ± SD. *p<0.05 test condition in the absence of BAY 11–7082 compared to test condition in the presence of BAY 11–7082.

We tested whether Mtb could reactivate latent HIV-1 using the JLAT 10.6 clone [26]. This cell line contains an integrated copy of HIV-1 and expresses GFP after HIV-1 reactivation from latency. The positive control, phorbol 12-myristate 13-acetate (PMA) activates protein kinase C to yield the expected expression of GFP (Fig 1A). In this cell line, very low responses were detected with the test agents (H37Ra or *Mycobacterium smegmatis* in co-culture, H37Rv lysate, PIM6, and LAM). It has been observed that Jurkat cells have low levels of TLR-2 expression and are insensitive to TLR-2 agonists [25, 30]. Therefore, in order to more efficiently test the specific role of TLR-2 in HIV-1 reactivation we stably transduced TLR-2 with a lentiviral vector to express high levels of TLR-2 (JLAT-TLR2) (S1 Fig). PIM6 and H37Rv lysate reactivated latent HIV-1 in the JLAT-TLR2 cells (Fig 1B). Whereas H37Ra, *M*. *smegmatis* and LAM did not cause significant reactivation in the JLAT-TLR2 cells. To confirm that the reactivation seen was indeed dependent on TLR-2 signaling, we tested the same conditions in the presence of a TLR-2 neutralizing antibody (PAb-hTLR2). Incubation with the TLR-2 neutralizing antibody significantly attenuated induction of GFP by PIM6 and H37Rv lysate (Fig 1C). A downstream mediator of TLR-2 activation is the transcription factor, NF-κB. Intriguingly, the HIV-1 LTR contains two NF-κB consensus binding sites within the enhancer region and NF-κB has been shown to be necessary for HIV-1 reactivation [31]. Thus, we tested the NF-κB antagonist, BAY 11–7082, for the ability block viral reactivation mediated by TLR-2. As expected, BAY 11–7082 attenuated viral reactivation mediated by PIM6 and H37Rv lysate (Fig 1D).

### PIM6, H37Rv lysate, and whole bacteria co-culture reactivate HIV-1 in a T_CM_ model of latency

**Fig 2:**
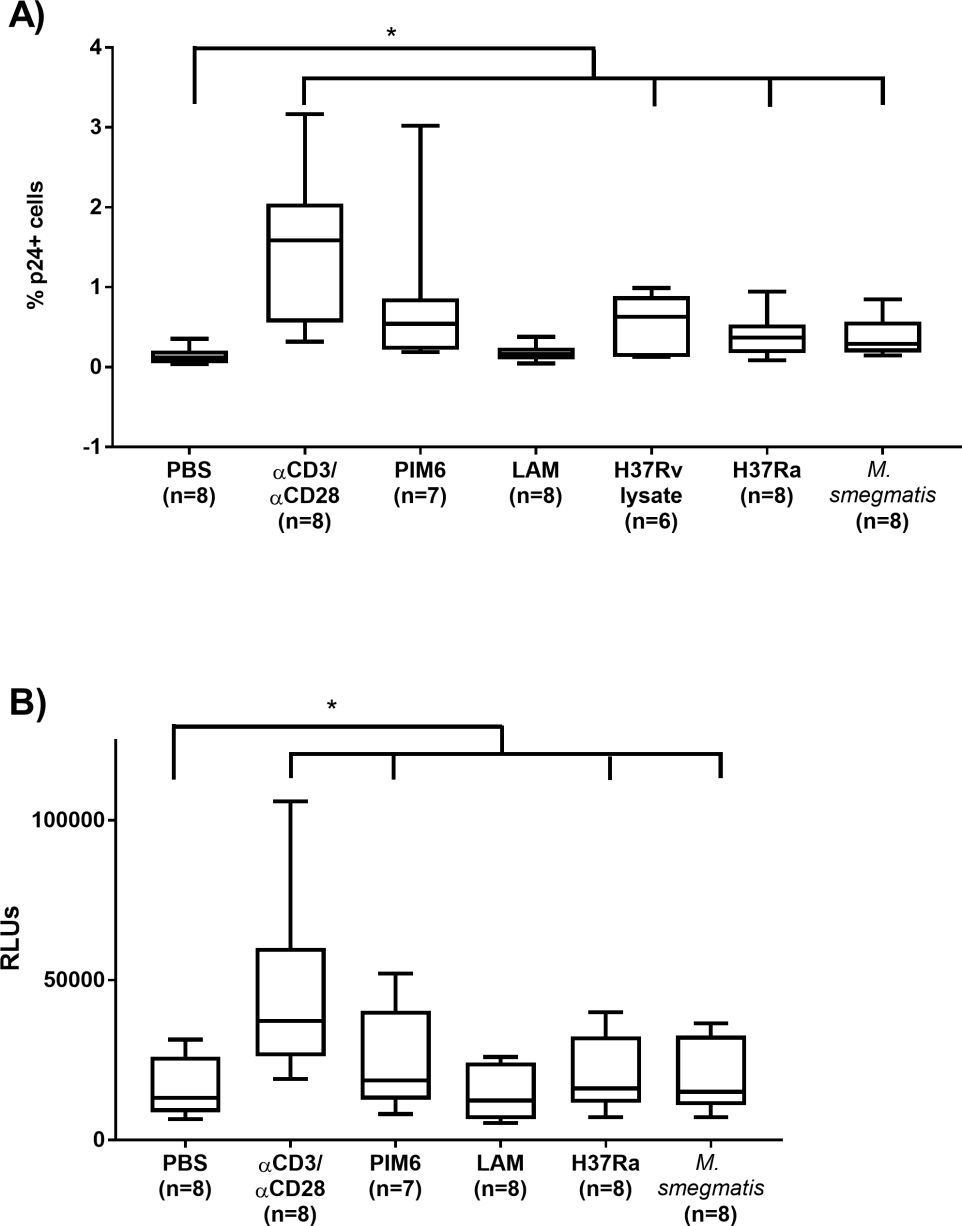
Reactivation of HIV by H37Ra, PIM6, and H37Rv lysate in a primary T_CM_ model of latency. Cultured T_CM_ cells following 72-hour incubation with test conditions or co-stimulation with αCD3/αCD28. (A) Levels of intracellular p24 Gag were measured by flow cytometry. The horizontal line within the box represents the median, the boundaries of the box represent the 25^th^- and 75^th^-percentile, and the whiskers represent the maximum and minimum values. Significance for intracellular p24 Gag was determined using a 2-tailed, paired Student’s t-test versus PBS (*p<0.05). (B) Relative luminescence was measured from supernatant of cultured T_CM_ cells following 72-hour incubation with conditions or co-stimulation with αCD3/αCD28. The horizontal line within the box represents the median, the boundaries of the box represent the 25^th^- and 75^th^-percentile, and the whiskers represent the maximum and minimum values. Significance was determined using a 2-tailed, paired Student’s t-test versus PBS (*p<0.05). Significance of individual test conditions are as follows: αCD3/αCD28 (p≤0.01), PIM6 (p<0.05), H37Ra (p≤0.01), and *M*. *smegmatis* (p≤0.01).

We sought to confirm the observations obtained with the JLAT model in a primary cell model of latency that uses cultured T_CM_ cells as targets for replication-competent HIV-1 [29]. For the work presented here, a slight modification was made to the published model; we utilized a replication-competent HIV-1 construct bearing the sec*NLuc* gene within the *nef* locus of HIV-1_NL4-3_. Cells latently infected with this modified, replication-competent HIV-1 secrete NanoLuc® luciferase enzyme into cell culture supernatant when treated with latency reversal agents. This allows for the ability to quantify HIV-1 reactivation from the supernatant of test cultures, providing timely detection of HIV-1 reactivation combined with the sensitivity of luminescence. We also measured intracellular p24 Gag production by flow cytometry. By measuring p24 Gag production, the results documented a statistically significant increase in reactivation in the whole bacteria co-culture conditions (H37Ra and *M*. *smegmatis*) and H37Rv lysate, and a trending increase PIM6 (due to inter-individual variation; Fig 2A). However, when luciferase activity was used to measure HIV-1 reactivation in these experiments, statistically significant increases in reactivation was observed with all the tested stimuli except LAM (Fig 2B). H37Rv lysate was observed to suppress luminescence even though latency reversal was indicated by increased p24 Gag (S3A & S3B Fig). We tested if this observation could be the result of H37Rv lysate interactions with the NanoLuc® enzyme or inhibition of enzyme secretion. Incubation of H37Rv lysate with supernatant from SupT1 cells infected with the modified HIV-1 showed attenuated luminescence (S3C Fig). Thus, we concluded that the observed suppression of luminescence is due to an undefined interaction with the NanoLuc® enzyme.

## Discussion

A major concern of HIV-1/Mtb co-infection in patients is accelerated HIV-1 infection [6]. While many studies provide evidence for increases in viral spread, mechanisms underlying the impact of Mtb on latent HIV-1 reservoirs have not been investigated. In our study, we found that whole *Mycobacteria* as well as their membrane component PIM6 and whole H37Rv lysate, which is composed of a mixture of proteins, lipids and carbohydrates from the bacterial cell, reversed HIV-1 latency. This was demonstrated using a T cell leukemia model of latency, JLAT 10.6 T cell line, and a primary human T_CM_ cell model of HIV-1 latency. The JLAT cell line is a stably HIV-1-infected Jurkat cell line expresses GFP upon activation of HIV-1 LTR transcription, and was developed by Jordan and colleagues to help enable understanding of HIV-1 latency [26]. JLAT cells readily permit more mechanistic experiments and are extremely amenable to genetic manipulation. Thanks to this ability, we were able to generate the TLR-2 overexpresser line used in our study. CD4^+^ T_CM_ cells are thought to be an important reservoir of latent HIV-1 in patients [32–36]. *In vitro* cultured T_CM_ cells faithfully recapitulate the biology of their *in vivo* counterparts and efficiently harbor latent infections [27]. The cultured T_CM_ cells reflect variations in signaling pathways found in patient populations.

Mycobacteria and some of their components were shown early on to induce HIV-1 LTR expression in human monocytes and other cell lines [20, 37–41]. Work in 1994 by Shattock and colleagues concluded that “phagocytosis of *M*. *tuberculosis* by monocytes expressing latent or restricted HIV-1 replication has the potential to induce or enhance viral replication *in vivo*” [39]. We recently confirmed that concurrent co-infection of THP-1 macrophages by Mtb and HIV-1 is achievable in the lab setting, but only demonstrated active replication of HIV-1in the presence of intracellular Mtb [42]. Toossi and colleagues, in 1999, took the investigations farther by showing increased production of HIV-1 transcription and viral release when HIV-1 infected monocytes from healthy donors were co-incubated with Mtb or Mtb protein components [43]. Several groups showed this induction to be dependent on TLR-2 and to be mediated, at least in part, by NF-κB binding to the HIV-1 LTR region [15, 25, 41, 43].

In addition to the mycobacteria, several other PAMP presenting organisms have been reported to transactivate the HIV-1 LTR via TLRs 5, 7/8 or 9 [44–46]. TLR-2 can form heterodimers with TLR-1 or TLR-6 to recognize many mycobacterial components including glycolipids, triacylated or diacylated lipopeptides [47, 48]. TLR-2 can also cooperate with TLR-10 and Dectin-1 [49, 50]. Interestingly, HIV-1 infection of monocyte-derived macrophages and peripheral blood mononuclear cells has been observed to up-regulate TLR-2 and TLR-4 expression [51]. This finding may indicate that HIV-1 could utilize PRRs as a self-amplification loop for viral reactivation.

Phagocytosis of mycobacteria by macrophages results in exosome release of lipids and other pro-inflammatory bacterial components that are capable of binding PRRs and activating T cells [52–56]. PIM6 is an example of such a mediator. PIM6 is a powerful TLR-2 agonist shown by Rodriguez and colleagues to up-regulate HIV-1 replication in productively infected and CD3 co-stimulated T cells [20]. Here we report that PIM6-mediated activation of TLR-2 can also lead to HIV-1 latency reversal.

Using the intracellular p24 Gag endpoint to quantify HIV-1 reactivation in the T_CM_ model, we were able to show statistically significant latency reversal in cultures co-incubated with H37Ra, *M*. *smegmatis*, and H37Rv lysate. This confirmed the basic hypothesis that the presence of Mtb in proximity to infected T cells is sufficient to reactivate latent HIV-1. While the other stimuli, such as PIM6, showed a trend in the induction of p24 Gag production, the variability observed using latently infected primary cell culture prevented further conclusions regarding its effects. However, when luciferase activity, a more sensitive endpoint than p24 expression, was used it became clear that, in addition to co-culture with whole *Mycobacteria* and H37Rv lysate, PIM6 significantly reversed HIV-1 latency in this model. These data support the idea that mycobacteria and mycobacterial membrane components can drive HIV-1 activation in latently infected T cells.

Experiments in JLAT cells interrogated the hypothesized major pathway of HIV-1 reactivation by Mtb. JLAT cells, while responding robustly to the positive control PMA activation, did not show statistically significant GFP induction with any of the test conditions. However, when TLR-2 was expressed ectopically, both PIM6 and H37Rv lysate were found to induce significant GFP expression. We found that TLR-2 neutralizing antibody, while not affecting the response to PMA, attenuated the induction responses of PIM6 and H37Rv lysate. Furthermore, use of the NF-κB antagonist, BAY 11–7082, also attenuated the GFP induction by PIM6 and H37Rv lysate. These data suggest that HIV-1 reactivation by PIM6 and H37Rv lysate in the T_CM_ model occurs through activation of the TLR-2 pathway. Although whole bacteria co-culture (H37Ra and *M*. *smegmatis*) did not significantly reactivate latent HIV-1 in the JLAT or JLAT-TLR2 studies, they were significant in our primary T_CM_ HIV-1 latency model. This indicates that pathways activated in our T_CM_ cells may vary in expression from those in the JLATs.

Interactions between Mtb and latent HIV-1 infected cells have remained largely undefined, yet HIV-1/Mtb co-infection is one of the world’s most lethal conditions. Our study suggests that chronic Mtb infection could drive latency reversal in T_CM_ cells, which may contribute to higher viral load, increased T cell loss and thereby T cell exhaustion. Even periodic infections by other microbial pathogens that activate TLR-2 or converging on pro-inflammatory signaling pathways (e.g. NF-κB) could conceivably contribute to transiently elevated levels of viremia in HIV-1 patients [25]. In poorly managed or non-compliant patients this could have negative health outcomes [57, 58]. While our report provides the initial observation, further work is needed to fully describe mechanisms and pathways by which mycobacteria might affect HIV-1 latency reversal.

## Materials & methods

### Reagents

The following reagents were obtained through BEI Resources, NIAID, NIH: *Mycobacterium tuberculosis*, Strain H37Rv, Purified Phosphatidylinositol Mannoside 6 (PIM6), NR14847; *Mycobacterium tuberculosis*, Strain H37Rv, Purified Lipoarabinomannan (LAM), NR-14848; *Mycobacterium tuberculosis*, Strain H37Rv, Whole Cell Lysate, NR-14822. The following bacteria were obtained from ATCC: *Mycobacterium tuberculosis* (Zopf) Lehmann and Neumann, Strain H37Ra, ATCC® 25177™; and *Mycobacterium smegmatis* (Trevisan) Lehmann and Neumann, ATCC® 607™. J-Lat Full Length cells 10.6 (JLAT) (Cat. #9849), Sup-T1 cells (Cat. #100) and nelfinavir (Cat. #4621) were obtained from the NIH AIDS Reagent Program. The 293FT cell line was obtained from Invitrogen™ (Cat. #R70007). Phorbol 12-myristate 13-acetate (PMA) was obtained from Fisher BioReagents (Cat. #BP685-1).

### Bacterial culture

H37Ra and *M*. *smegmatis* were grown to mid-to-late-logarithmic phase in Difco™ Middlebrook 7H9 Broth (Becton, Dickinson and Company; Cat. #271310) supplemented with ADC enrichment media (Remel™, ThermoFisher Scientific, Cat. #R450592), 0.2% glycerol and 0.05% Tween 80 at 37°C. For assays, bacterial cultures were adjusted to a total OD_600_ of 1.2 reconstituted in 1 mL of phosphate buffered saline (PBS) prior to addition to wells.

### Generation of stable TLR-2 expressing JLAT 10.6 cells

A bicistronic lentiviral vector derived from pFIN-EF1-GFP-2A-mCherry-WPRE (kindly provided by Dr. Susan L. Semple-Rowland, University of Florida McKnight Brain Institute) was engineered to express TLR-2 (https://www.ncbi.nlm.nih.gov/gene/7097) in place of GFP. The resulting lentiviral vector, pFIN-EF1-TLR2-2A-mCherry-WPRE, encodes a fusion of TLR-2 and mCherry, whose expression is driven by the elongation factor 1 (EF1) promoter (S1 Fig). The presence of the intervening 2A peptide from porcine teschovirus-1 leads to ribosomal skipping and equimolar production of TLR-2 and mCherry [59]. As a control, we also constructed a vector that only encodes the mCherry protein. J-LAT cells were infected with the lentiviral vectors and mCherry expressing cells were isolated by FACS.

### JLAT and JLAT-TLR2 assays

Cell lines were maintained in RPMI1640 media supplemented 10% fetal bovine serum (FBS) (Atlanta Biologics, Cat. #S11150) and antibiotic-antimycotic solution (100X, GIBCO, Grand Island, NY, USA) at 37°C, 5% CO_2_. JLAT and JLAT-TLR2 cells were plated at 100,000 cells/well in 96-well U-bottom culture plates and incubated with the following conditions for 16 hours: PBS, PMA (200 ng/mL), PIM6 (10 μg/mL), H37Rv lysate (100 μg/mL), LAM (100 μg/mL), H37Ra (MOI 35:1) and *M*. *smegmatis* (MOI 35:1). For assays involving inhibitors, cells were pre-incubated for 30 minutes at 37°C in media containing PAb-hTLR2 antibody (20 μg/mL; InvivoGen, Cat. Code pab-hstlr2) or BAY 11–7082 (0.30 μM; EMD Millipore Calbiochem™, Cat. #19-687-010MG). Cells were stained for viability (BD Horizon™ Fixable Viability Stain 450) and fixed with 1% paraformaldehyde. Viability and GFP expression was measured using a BD FACS Canto.

### Primary cultured T_CM_ cell model of HIV-1 latency

Peripheral blood mononuclear cells were collected from unidentified, healthy donors following protocols outlined in IRB #67637 (University of Utah Institutional Review Board approved). Naïve CD4^+^ T cells were isolated using an immunomagnetic negative selection kit (EasySep ™ Human Naïve CD4^+^ T Cell Isolation Kit, STEMCELL™ Technologies, Cat. #19555). Following isolation, cells were activated with human αCD3/αCD28-coated magnetic beads (1 bead/10^6^ cells, Life Technologies, Cat. #11131D), αIL-4 (2 μg/10^6^ cells, Peprotech, Cat. #500-p24), αIL-12 (4 μg/10^6^ cells, Peprotech, Cat. #500-p154g) and tumor growth factor (TGF)-β1 (0.8 μg/10^6^ cells, Peprotech, Cat. #100–21) for 3 days. Donor cells were subsequently infected at MOI 0.1 via spinoculation with HIV^Nluc2A^ (2,900 rpm for 2 hours at 37°C). Infected cells were expanded over 7 days. Viral replication was suppressed by incubating cells in nelfinavir (1 μM) for 16 hours at 37°C, 5% CO_2_. Prior to assay, HIV^Nluc2A^-infected CD4^+^ T cells were isolated (Dynabead CD4-positive isolation kit, Life Technologies, Cat. #11551D) upon which cells were resuspended in RPMI 1640 supplemented with 10% FBS, antibiotic-antimycotic solution, recombinant human IL-2 (30 IU/mL, Dr. Maurice Gately, Hoffman-La Roche Inc. [60]), and nelfinavir (1 μM). Cells were plated at a cell density of 50,000 cells/well in 96-well U-bottom plates. Cells were incubated with the following conditions for 72 hours at 37°C, 5% CO_2_ (in triplicate): PBS, αCD3/αCD28 beads (1 bead/10^6^ cells), PIM6 (10 μg/mL), H37Rv lysate (100 μg/mL), LAM (100 μg/mL), H37Ra (MOI 70:1) and *M*. *smegmatis* (MOI 70:1).

### Flow cytometry

TLR-2 surface expression was determined using anti-human CD282-APC (and isotype control (Biolegend, San Diego, CA)) Expression was measured in a BD FACSCanto II flow cytometer. For analysis of T_CM_ model, cells were stained with viability dye (Fixable Viability Dye efluor450, affymetrix, eBioscience, San Diego, CA) and mouse anti-human CD4-APC antibody (cloneS3.5, Invitrogen) for 30 minutes at 4°C followed by a fixation/permeabilization step (FIX & PERM® Cell Fixation & Cell Permeabilization Kit, ThermoFisher Scientific, Cat. #GAS004). Cells were stained for intracellular p24 Gag by incubating cells with ICp24-FITC antibody (KC57, Coulter) in BD Perm/Wash™ Buffer (BD Biosciences, Cat. #554723) for 30 minutes at 4°C. Conditions were analyzed on BD FACS Scan. Data were analyzed by FlowJo (TreeStar Inc., Ashland, OR).

## Generation of pNL43-secNLuc2Anef

A PCR product containing the sec*NLuc* gene flanked by 5’ NheI and 3’ BamHI restriction sites was generated using PCR primers (5’-ccctgctagcgtcttcacactcgaagatttcgttgggg and 5’-cgcgggatcccgccagaatgcgttcgcacagccg) and pNL1.3[sec*NLuc*], a plasmid containing the coding sequence for secreted NanoLuc® luciferase (Promega, Cat. #N1021). This PCR product and the plasmid pFIN-EF1-GFP-2A-HA-VPR-WPE were digested with NheI and BamHI. The digested secNLuc PCR fragment and the10.6 kb fragment from the EF1-GFP-2A-HA-VPR-WPE plasmid were ligated to generate pFIN-EF1-nLuc-2A-HA-vpr-WRE. A PCR product containing the first 152 nucleotides of coding sequence of the HIV-1 *nef* gene flanked by 5’ EcoRV and 3’ MluI was generated using PCR primers (5’-ttttgcgatatcatgggtggcaagtggtcaaaaagtagtgtgat and 5’-ttagctgctgtaacgcgtcttgtgattgctccatgtttttctaggtc) and pNL4-3. The *nef* PCR product and pFIN-EF1-secNluc-2A-HA-VPR-WRE plasmid were digested with EcoRV and MluI. The 10.8 kb fragment from the pFIN-EF1-secNluc-2A-HA-VPR-WRE plasmid and the EcoRV and MluI digested *nef* PCR product were ligated to create the pFIN-EF1-secNluc-2A-nef-WRE plasmid. An NcoI restriction site was introduced into pUC19 using site-directed mutagenesis (QuickChange Lightning, Agilent Genomics) and primers (5’-ggtacccggggatcccatggagtcgacctgcagg and 5’-cctgcaggtcgactccatgggatccccgggtacc) to generate the pUC19NcoI plasmid. The pUC19NcoI plasmid was digested with NcoI and SmaI. pNL4-3 was digested with NheI followed by incubation with the Klenow large fragment to fill in the overhang created by NheI digestion. The resulting linearized plasmid was digested with NcoI to create a 3318 bp fragment containing 1530 nucleotides of the 3’ coding sequence of HIV-1 *env*, the *nef* gene and the 3’ LTR. This fragment was ligated with the NcoI- and SmaI-digested fragment of pUC19NcoI (2678 bp) to create pUC19HIVNcoINheI. A unique XbaI site within the 5’ coding sequence of HIV-1 *nef* in pUC19HIVNcoINheI was generated using site-directed mutagenesis (QuickChange Lightning, Agilent Genomics) and primers (5’-ccatccaatcacactacttctagaccacttgccacccatct and 5’-agatgggtggcaagtggtctagaagtagtgtgattggatgg) to create pUC19HIVNcoINheIaddXbaI. The pUC19HIVNcoINheIaddXbaI plasmid was digested with XbaI and XhoI, a unique restriction site within the HIV-1 *nef* gene. The pFIN-EF1-secNluc-2A-nef-WRE plasmid was digested with NheI and XhoI to generate a 778 bp fragment containing secNluc, 2A and 106 bp of the 5’ coding sequence of the HIV-1 *nef* gene. The 778 bp nLuc-2A-5’*nef* fragment was ligated to the 5911 bp XbaI XhoI fragment from pUC19HIVNcoINheIaddXbaI to replace 85 bp of coding sequence within the HIV-1 *nef* gene with secNluc-2A-5’nef. This reconstitutes the *nef* gene through ligation of 5’ and 3’ termini at the native XbaI restriction site. The resulting plasmid, named pUC19-secNluc2Anef, and pNL4-3 were digested with HpaI and NcoI. The 2611 bp fragment from pUC19-secNluc2Anef and the 12961 bp fragment from pNL4-3 were ligated to create pNL43-secNluc2Anef (S4 Fig).

### Generation of HIV^NLuc2A^ virus by calcium phosphate transfection

293FT cells were grown to approximately 70% confluency in 150 cm^2^ cell culture dishes. A CaPO_4_-DNA suspension of pNL43-secNluc2Anef and pCMV-VSVg (approximately 25 μg each) was prepared by diluting in ddH_2_O and subsequently mixing with CaCl_2_ (2.5 M) and 2X HBS (pH 7.05). CaPO_4_-DNA suspension was added drop-wise to culture dishes followed by the addition of chloroquine (100 mM). Cells were incubated for 16 hours and media was replaced. Supernatant was recovered after 48 hours and sterile-filtered (0.45 μM). Viral titers were determined using Sup-T1 cells.

### Nanoluciferase assay

Ten microliter aliquots of the supernatant from T_CM_ experiment wells were mixed with 0.1% bovine serum albumin (BSA) in PBS. Nano-Glo® Luciferase Assay Substrate (Promega) was diluted 1/50 in Nano-Glo® Luciferase Assay Buffer (Promega). The mixture was incubated at room temperature for 5 min and luminescence was quantified using a Biotek Synergy 2 microplate reader.

### Experimental and statistical analysis

Cell-line based assays (JLAT & JLAT-TLR2) were conducted in triplicate and mean values were calculated across four and ten independent experiments, respectively. Significance was determined using an unpaired, two-tailed Student’s t-test versus PBS. For inhibitor-based assays, three independent experiments ran in triplicate were performed and mean values were calculated. An unpaired, two-tailed Student’s t-test comparing test conditions in the absence and presence of inhibitor was used to determine significance. The T_CM_ model was run in triplicate across eight donors with the exception of PIM6 (n = 7) and H37Rv lysate (n = 5). Analysis of flow cytometry data for the T_CM_ model was conducted for the mean percent p24 positive values for eight donors. Significance was determined using a paired, two-tailed Student’s t-test versus PBS. For the NanoLuc® luciferase assay, the mean relative luminescence units for each donor was calculated and significance was determined using a paired, two-tailed Student’s t-test versus PBS. All statistical analyses were performed using GraphPad Prism (GraphPad Software, Inc., La Jolla, CA).

### Ethics statement

PBMCs were collected from unidentified, healthy donors following protocols outlined in IRB #67637 (University of Utah Institutional Review Board approved). All donors provided written informed consent prior to entry to the study.

## Supporting information

S1 Fig TLR-2 overexpression in JLAT 10.6 cells. A) The lentiviral vector, pFIN-EF1-GFP-2A-mCherry-WPRE, was engineered to express TLR-2 in place of GFP. B) Anti-human CD282 (open black histogram) and isotype control (closed gray histogram) were used to determine surface expression of TLR-2.
(TIF)

S2 Fig Reactivation of HIV by H37Ra, PIM6, and H37Rv lysate in a primary T_CM_ model of latency. Cultured T_CM_ cells following 72-hour incubation with test conditions or co-stimulation with αCD3/αCD28. (A) Levels of intracellular p24 Gag were measured by flow cytometry. Each symbol corresponds to a different donor. Mean ± SD are indicated with the horizontal lines.). (B) Relative luminescence was measured from supernatant of cultured T_CM_ cells following 72-hour incubation with conditions or co-stimulation with αCD3/αCD28. Each symbol corresponds to a different donor. Mean ± SD are indicated with the horizontal lines. Significance was determined using a 2-tailed, paired Student’s t-test versus PBS (*p<0.05). Significance of individual test conditions are as follows: αCD3/αCD28 (p≤0.01), PIM6 (p<0.05), H37Ra (p≤0.01), and *M*. *smegmatis* (p≤0.01).
(TIF)

S3 Fig H37Rv lysate induces p24 expression but suppresses luminescence in a primary T_CM_ model of latency. Cultured T_CM_ cells following 72-hour incubation with test conditions or co-stimulation with αCD3/αCD28. (A) Levels of intracellular p24 Gag were measured by flow cytometry. The horizontal line within the box represents the media, the boundaries of the box represent the 25^th^- and 75^th^-percentile, and the whiskers represent the maximum and minimum values. Significance for intracellular p24 Gag was determined using a 2-tailed, paired Student’s t-test versus PBS (*p<0.05). (B) Relative luminescence was measured from supernatant of cultured T_CM_ cells following 72-hour incubation with conditions or co-stimulation with αCD3/αCD28. The horizontal line within the box represents the media, the boundaries of the box represent the 25^th^- and 75^th^-percentile, and the whiskers represent the maximum and minimum values. Significance was determined using a 2-tailed, paired Student’s t-test versus PBS (*p<0.05). (C) Supernatant from SupT1 cells infected with HIV^Nluc2A^ (MOI 0.1) or supernatant from uninfected SupT1 cells (Mock) was incubated with H37Rv lysate (100 μg/mL) for 72 hours at 37°C after which point luminescence was measured. Significance was determined using a one-tailed, unpaired Student’s t-test (p<0.05).
(TIF)

S4 Fig Plasmid map of pNL43-secNluc2Anef. (TIF)
