# Supplementary figures and images for: Mycobacterium tuberculosis reactivates latent HIV-1 in T cells in vitro
Source: PLoS One. 2017 Sep 26;12(9):e0185162. doi: 10.1371/journal.pone.0185162 (PMC5614573; doi:10.1371/journal.pone.0185162)

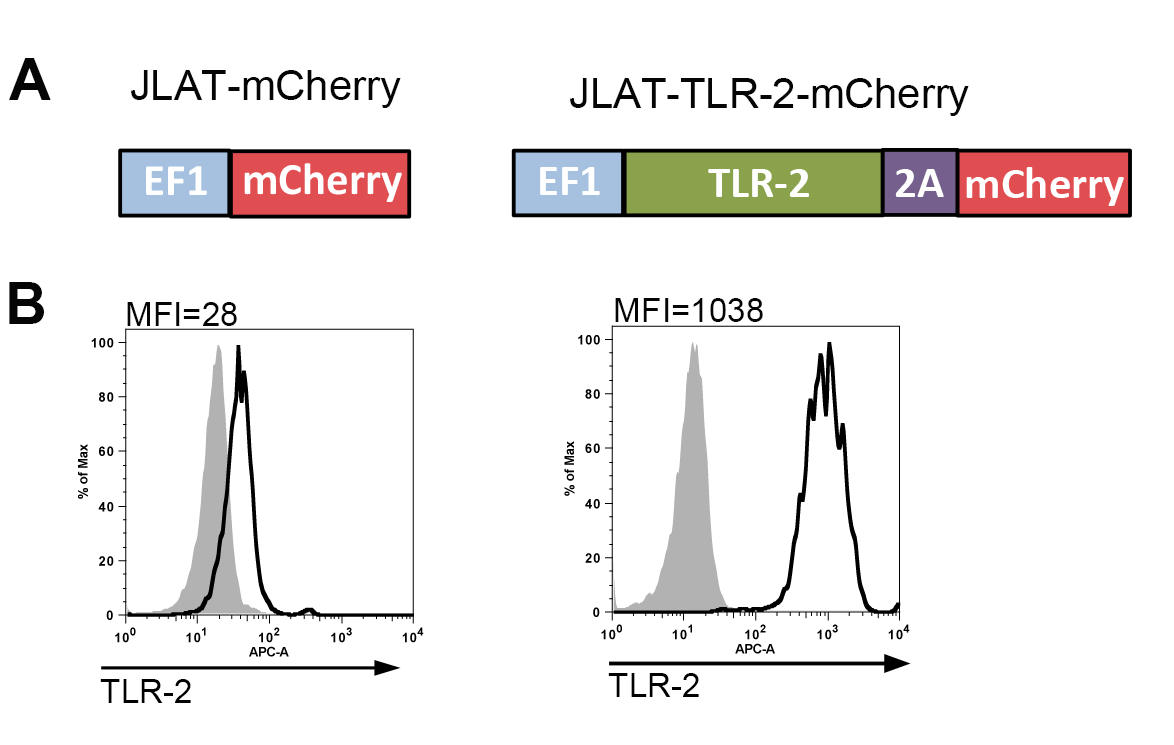

Supplement: S1 Fig — A) The lentiviral vector, pFIN-EF1-GFP-2A-mCherry-WPRE, was engineered to express TLR-2 in place of GFP. B) Anti-human CD282 (open black histogram) and isotype control (closed gray histogram) were used to determine surface expression of TLR-2. (TIF) [file pone.0185162.s001.tif]

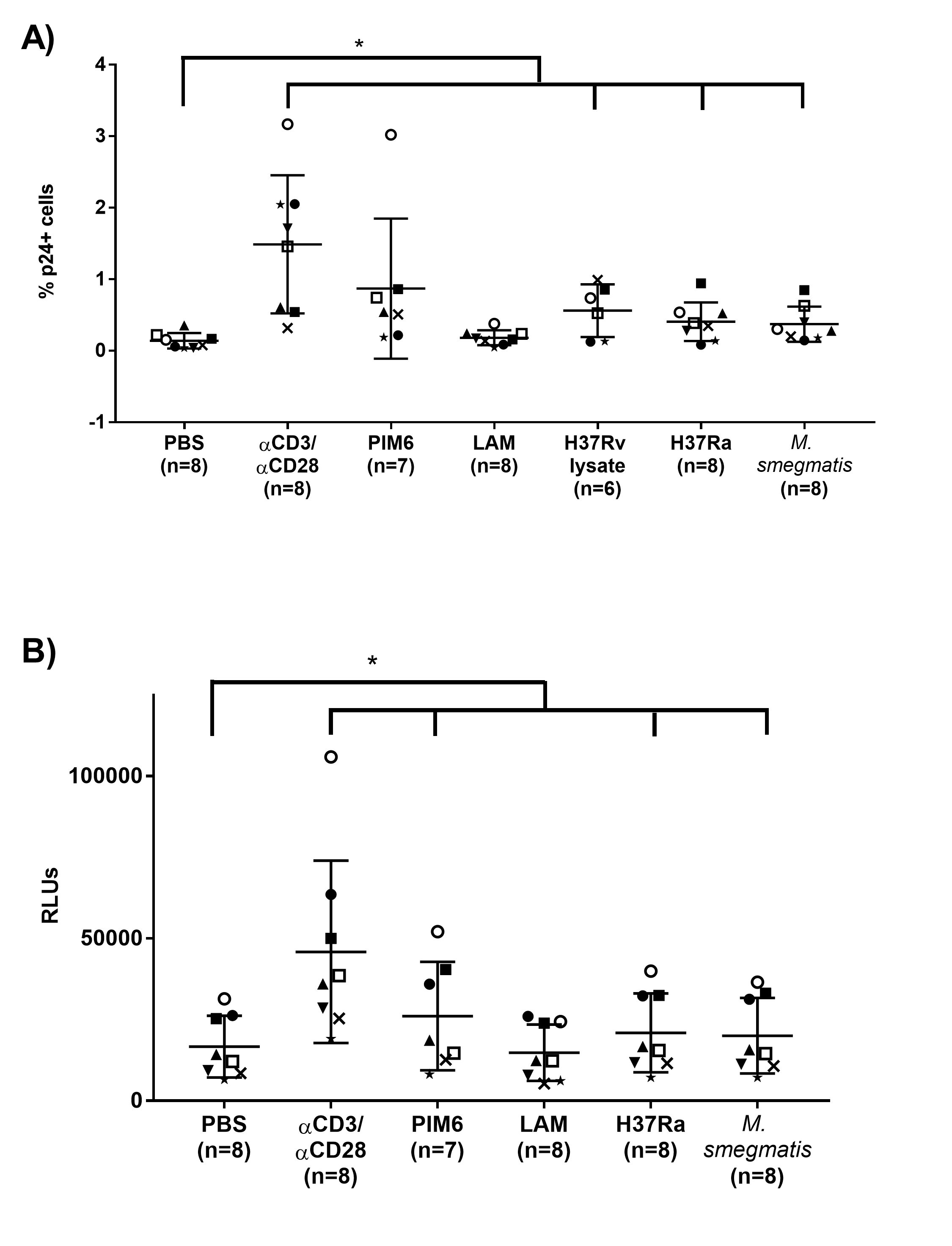

Supplement: S2 Fig — Cultured TCM cells following 72-hour incubation with test conditions or co-stimulation with αCD3/αCD28. (A) Levels of intracellular p24 Gag were measured by flow cytometry. Each symbol corresponds to a different donor. Mean ± SD are indicated with the horizontal lines.). (B) Relative luminescence was measured from supernatant of cultured TCM cells following 72-hour incubation with conditions or co-stimulation with αCD3/αCD28. Each symbol corresponds to a different donor. Mean ± SD are indicated with the horizontal lines. Significance was determined using a 2-tailed, paired Student’s t-test versus PBS (*p<0.05). Significance of individual test conditions are as follows: αCD3/αCD28 (p≤0.01), PIM6 (p<0.05), H37Ra (p≤0.01), and M. smegmatis (p≤0.01). (TIF) [file pone.0185162.s002.tif]

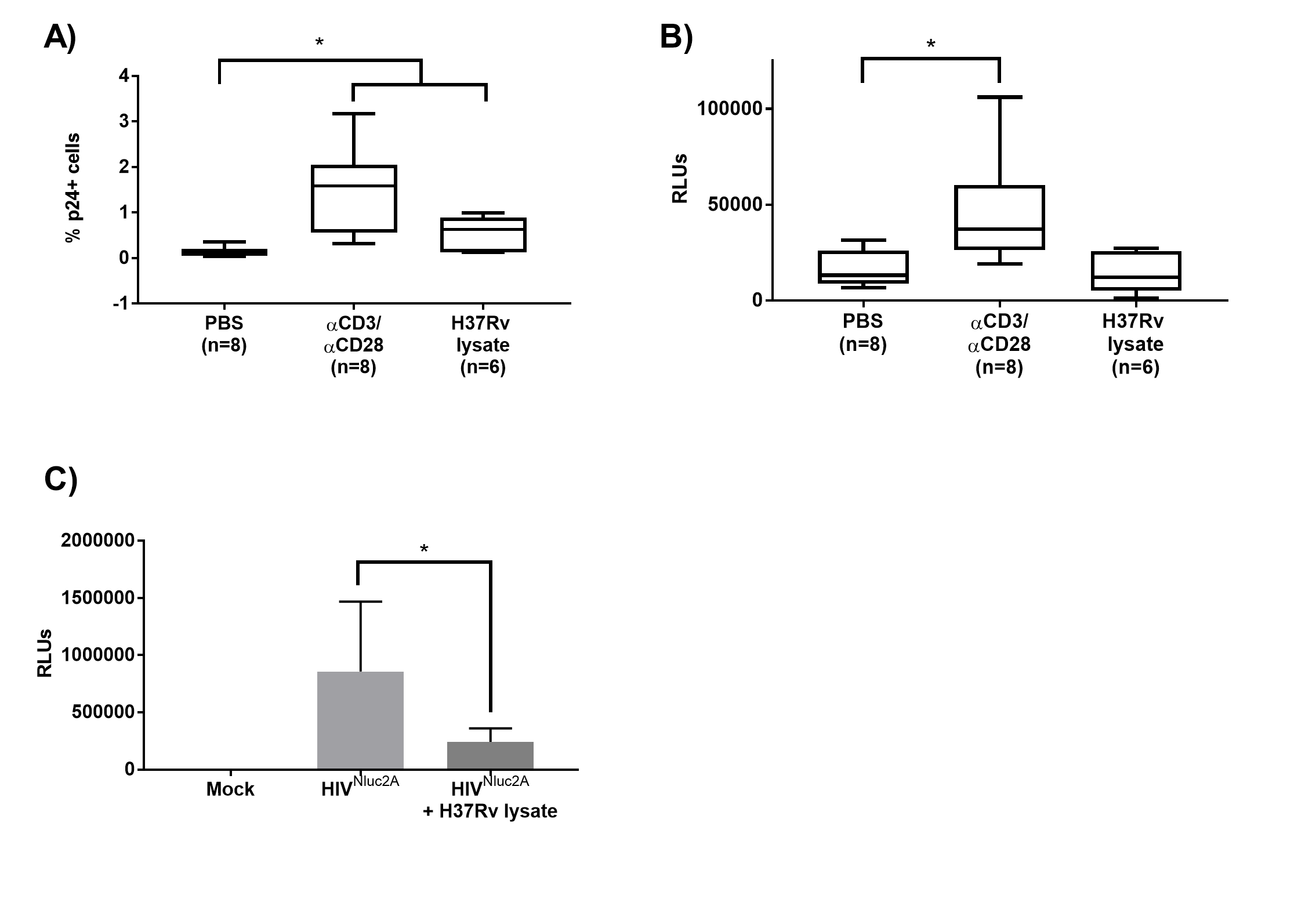

Supplement: S3 Fig — Cultured TCM cells following 72-hour incubation with test conditions or co-stimulation with αCD3/αCD28. (A) Levels of intracellular p24 Gag were measured by flow cytometry. The horizontal line within the box represents the media, the boundaries of the box represent the 25th- and 75th-percentile, and the whiskers represent the maximum and minimum values. Significance for intracellular p24 Gag was determined using a 2-tailed, paired Student’s t-test versus PBS (*p<0.05). (B) Relative luminescence was measured from supernatant of cultured TCM cells following 72-hour incubation with conditions or co-stimulation with αCD3/αCD28. The horizontal line within the box represents the media, the boundaries of the box represent the 25th- and 75th-percentile, and the whiskers represent the maximum and minimum values. Significance was determined using a 2-tailed, paired Student’s t-test versus PBS (*p<0.05). (C) Supernatant from SupT1 cells infected with HIVNluc2A (MOI 0.1) or supernatant from uninfected SupT1 cells (Mock) was incubated with H37Rv lysate (100 μg/mL) for 72 hours at 37°C after which point luminescence was measured. Significance was determined using a one-tailed, unpaired Student’s t-test (p<0.05). (TIF) [file pone.0185162.s003.tif]

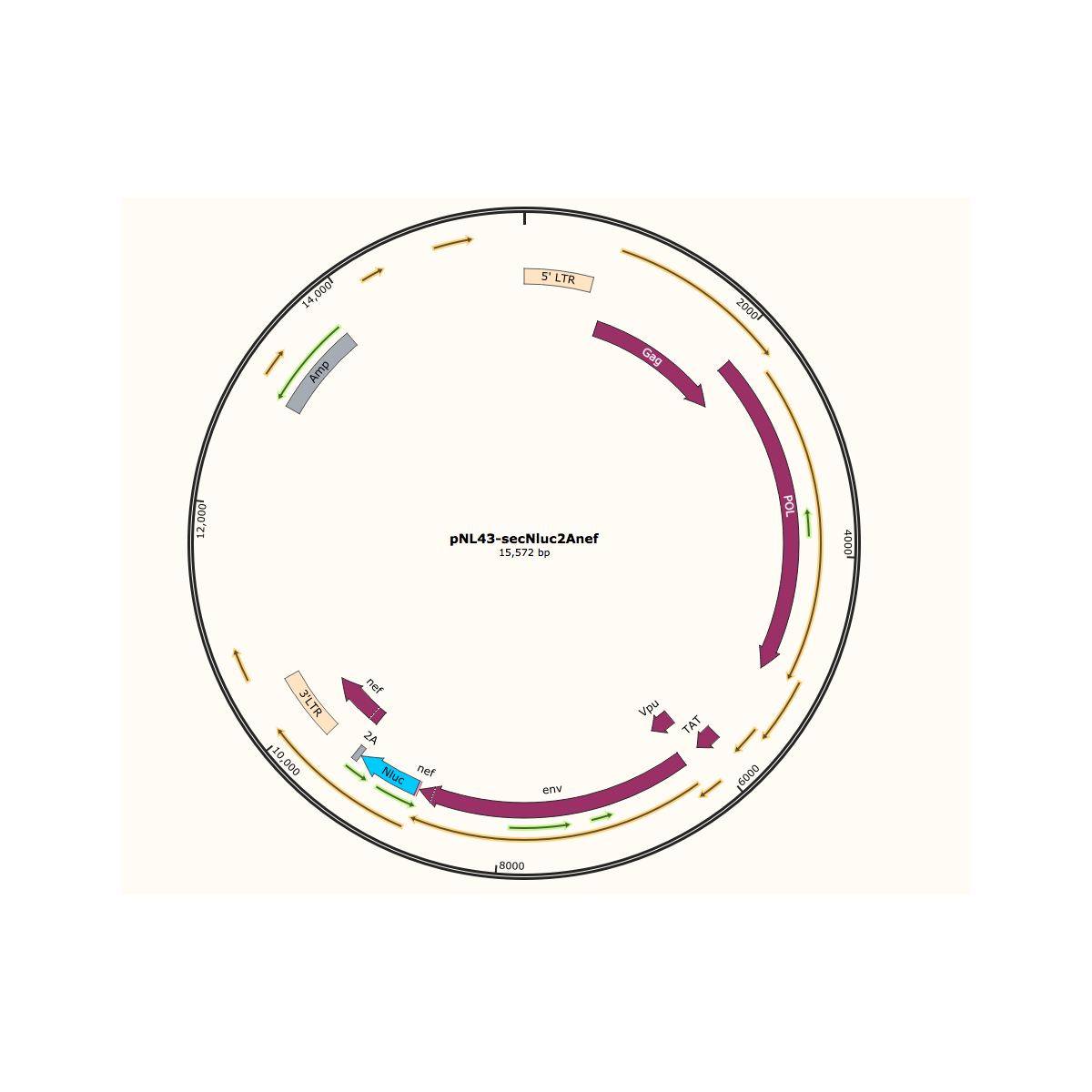

Supplement: S4 Fig — (TIF) [file pone.0185162.s004.tif]
